# Genetic diversity of *BoLA-DRB3* in Latin American Creole cattle: an update of the state of the art

**DOI:** 10.1007/s00251-025-01384-w

**Published:** 2025-09-29

**Authors:** Olivia Marcuzzi, Guillermo Giovambattista, Ariel Loza Vega, Juan Antonio Pereira Rico, Maria Florencia Ortega Masague, Liz Aurora Castro Rojas, Ruben Martinez, Odalys Uffo Reinosa, Aronggaowa Bao, Sonoko Watanuki, Noriko Fukushi, Fumihiro Nagata, Ryosuke Matsuura, Yoko Aida

**Affiliations:** 1 Facultad de Ciencias Veterinarias UNLP, IGEVET-Instituto de Genética Veterinaria “Ing. Fernando N. Dulout” (UNLP-CONICET LA PLATA), 1900 La Plata, Argentina; 2 Laboratory of Global Infectious Diseases Control Science, Graduate School of Agricultural and Life Sciences, The University of Tokyo, 1-1-1 Yayoi, Bunkyo-Ku, Tokyo, 113-8657 Japan; 3 Facultad de Ciencias Veterinarias, Universidad Autónoma Gabriel René Moreno, Santa Cruz de La Sierra, Bolivia; 4 Agencia de Extensión Rural (AER Lules- EEA Famaillá), Instituto Nacional de Tecnología Agropecuaria (INTA), Famaillá, Tucumán, Argentina; 5 Facultad de Ciencias Veterinarias, Universidad Nacional de Asunción, San Lorenzo, Paraguay; 6 Facultad de Ciencias Agrarias, Universidad Nacional de Lomas de Zamora, Lomas de Zamora, Argentina; 7 Centro Nacional de Sanidad Agropecuaria (CENSA), San José de Las Laja, La Habana, Cuba

**Keywords:** Major histocompatibility complex class II, *BoLA-DRB3* polymorphism, Genetic variation, Native cattle, Principal component analysis, Population tree

## Abstract

Genetic diversity of the bovine leukocyte antigen *BoLA-DRB3* gene has been reported in cattle breeds due to its central role in immune response. However, the gene has been characterized in only a few Latin American native cattle breeds. In this study, we aimed to investigate the genetic diversity of this gene in other native bovine breeds from Argentina, Bolivia, Cuba, and Paraguay, thereby contributing to the global knowledge regarding *BoLA-DRB3* diversity. The gene was genotyped using PCR-sequence-based typing; 60 reported alleles and one novel variant were identified. The observed and expected heterozygosity values were higher than 0.81, and only two populations showed deviation from the Hardy–Weinberg equilibrium. The Slatkin neutrality test indicated even allele distribution in one population. Nucleotide diversity and the number of pairwise differences ranged from 0.073 to 0.081 and 17.88 to 20.19, respectively. The mean number of non-synonymous and synonymous nucleotide substitutions was estimated for the entire sequence and the antigen-binding site (ABS), with higher values observed in the latter. Amino acid motifs varied in their frequencies, and the diversifying selection index (ω) at each amino acid was high in more than 25 sites, with the most prominent peaks located in the ABS. A distribution of native cattle according to their historical and geographical origin and degree of Zebu gene introgression demonstrated that the Latin American native populations exhibit high variability in the *BoLA-DRB3* gene, including private alleles, and a distinct genetic profile, making them a valuable genetic resource and emphasizing the importance of their conservation.

**Supplementary Information:**

The online version contains supplementary material available at 10.1007/s00251-025-01384-w.

## Introduction

 The major histocompatibility complex (MHC) is an essential component of the adaptive immune system. Its primary function is to present peptides from host- and pathogen-expressed proteins on the cell surface for T-cell recognition (Klein 1986; Wieczorek et al. 2017). In cattle, the MHC system is known as bovine leukocyte antigen (*BoLA*), and is divided into three distinct regions, namely class I, class II, and class III, located on chromosome 23. Class I genes encode proteins that present intracellular peptides to cytotoxic T cells (CD8^+^), while class II genes encode proteins that present antigen peptides to CD4^+^ T cells (Takeshima and Aida 2006). A major reorganization within class II resulted in the division of *BoLA* into two sub-regions, namely class IIa and class IIb (Aida et al. 2015). The high levels of polymorphism in the MHC of both wild and domestic populations and variation in allele frequencies of *BoLA* class II genes across breeds are of great importance to evolutionary biologists. In cattle, class IIa includes multiple *DR* and *DQ* genes, which are extremely polymorphic, particularly at residues within the peptide-binding groove (Brown et al. 1993). These genes have been linked to immune responses in a variety of infectious diseases (Sharif et al. 1999; Rothschild et al. 2000; Takeshima and Aida 2006). Particularly, the *BoLA-DRB3* gene affects the magnitude and epitope specificity of antigen-specific T-cell responses and is the most polymorphic class II locus (Aida 1995; Takeshima et al. 2015a, b). *BoLA-DRB3* alleles have been associated with resistance or susceptibility to infectious diseases (e.g., bovine leukemia virus-induced lymphocytosis, mastitis, dermatophilosis, anaplasma, and babesia), as well as immunological and production traits and vaccine responses (Takeshima and Aida 2006; Yoshida et al. 2009; Chaudhary et al. 2022; Casa et al. 2023; Hamada et al. 2023).

Till date, the *BoLA-DRB3* gene has been characterized in only a few Latin American local cattle breeds (Giovambattista et al. 2013, 2020b; Bohorquez et al. 2020; Valenzano et al. 2022). In this study, we aimed to investigate the genetic diversity of this gene in other native bovine breeds from Argentina, Bolivia, Cuba, and Paraguay. Our results were compared with the published data to provide a comprehensive interpretation and contribute to the global knowledge regarding *BoLA-DRB3* diversity. This approach could possibly contribute to the following hypotheses: Latin American native populations exhibit a high degree of genetic variability and a distinct profile of the *BoLA-DRB3* gene, including private variants, due to their origin and subsequent adaptation; Creole populations from the same country may have diverged as a result of environmental adaptation (e.g., Highland Bolivian Creole [HBC] vs. Lowland Bolivian Creole [LBC], and Argentine Creole [CrAr] vs. Patagonian Argentine Creole [CrArPat]); the composite breed Siboney from Cuba either maintained the theoretical proportion of $^{{~}^{5}\!\left/ \!\!{~}_{8}\right.}$ Holstein-$^{{~}^{3}\!\left/ \!\!{~}_{8}\right.}$ Zebu or experienced allele enrichment from one parent due to adaptation to tropical conditions.

## Materials and methods

### Animal samples

Blood samples were collected from 238 adult cattle from six breeds, including CrAr (*N* = 107), CrArPat (*N* = 22), Bolivian Creole from Cochabamba Department (CrCoch, *N* = 13), Bolivian Saavedreño Creole (CrSaa, *N* = 10), Paraguayan Pampa Chaqueño Creole (PaCh, *N* = 36), and the Siboney composite breed from Cuba (SibCu, *N* = 50) (Table S1 and Fig. S1). Additionally, previously published data from other cattle breeds were included in this study. These samples comprised Creole breeds: Bolivian Creole from Oruro Department (CrAl, *N* = 48), Bolivian Yacumeño (CrYa, *N* = 112), Harton del Valle from Colombia (HaVa, *N* = 66), Lageano from Brazil (CrLag, *N* = 208); Taurine European breeds: Angus (AA, *N* = 100), Hereford (He, *N* = 49), Shorthorn (Sho, *N* = 100), Holstein from Argentina (HoAr, *N* = 424), Bolivia (HoBo, *N* = 159), and Paraguay (HoPar, *N* = 127); and Zebu breeds: Gir (Gir, *N* = 110), Nelore (Ne, *N* = 116), and Nelore-Brahman crossbreed (NeBh, *N* = 195) (Giovambattista et al. 2013, 2020b; Takeshima et al. 2015a, b, 2018; Casa et al. 2023; Table S1 and Fig. S1). Bolivian Creole cattle populations were grouped according to their geographical environments, namely HBC for those living at altitudes above 3000 m (CrCoch and CrAl) and LBC for those raised in the western plains (CrSaa and CrYa), resulting in a total of 8 Latin American cattle populations (CrAr, CrArPat, HBC, LBC, PaCh, SibCu, CrLag, and HaVa). In this study, the terms breed and population have been used as synonyms, considering the population as a comprehensive term that includes well-defined breeds as well as groups of animals that have not yet been defined as a breed (Ajmone-Marsan et al. 2023).

### *BoLA-DRB3* typing

*BoLA-DRB3* alleles were genotyped using PCR-sequence-based typing (PCR-SBT), as described by Takeshima et al. (2011). Exon 2 of the *BoLA-DRB3* was amplified using the primers DRB3FRW and DRB3REV (Baxter et al. 2009) (Table S2). The PCR fragments were then sequenced using the ABI PRISM BigDye Terminator Cycle Sequencing Ready Reaction Kit (Applied Biosystems, Foster City, CA). The raw sequencing data were analyzed using Assign 400ATF ver.1.0.2.41 software (Conexio Genomics, Fremantle, Australia).

### Gene frequency and genetic diversity at allele level

Allele frequencies, the number of alleles (n_a_), observed heterozygosity (h_o_), and unbiased expected heterozygosity (h_e_) for the *BoLA-DRB3* locus were estimated using Arlequin v3.5 software (Excoffier and Lischer 2010). Hardy–Weinberg equilibrium (HWE) was assessed using F-statistics (Weir and Cockerham 1984) for each breed with the exact test implemented in Genepop 4.7 software (Rousset 2008). The Ewens–Watterson–Slatkin exact test of neutrality was conducted using the Slatkin method (Slatkin 1996) in Arlequin v3.5. To determine whether the observed gene frequencies in the composite breed SibCu aligned with the expected theoretical values, the following formula was used:

$${{\Sigma (\mathrm{p}}_{\mathrm{i}}}_{\mathrm{Sib}} - {{(\mathrm{m}}_{\mathrm{Ho}}\times {{\mathrm{p}}_{\mathrm{i}}}_{\mathrm{Ho}}\hspace{0.17em}+\hspace{0.17em}{\mathrm{m}}_{\mathrm{Ne}} \times {\mathrm{p}}_{\mathrm{iNe}}))}^{2}$$

where p_iSib_ is the gene frequency of *BoLA-DRB3* in the composite population, p_iHo_ and p_iNe_ are the gene frequencies in the parent populations, respectively, and m_Ho_ and m_Ne_ are the theoretical proportions of genes from Holstein and Ne populations ($^{{~}^{5}\!\left/ \!\!{~}_{8}\right.}$ and $^{{~}^{3}\!\left/ \!\!{~}_{8}\right.}$, respectively).

### Genetic diversity at DNA and amino acid sequence levels

Genetic diversity was examined at the DNA level using nucleotide diversity (π) and the number of pairwise differences (NPD), estimated with Arlequin v3.5. Pairwise comparisons of nucleotide substitutions between alleles were calculated as the average number of differences between pairs of DNA sequences. The mean number of nonsynonymous (d_N_) and synonymous (d_S_) nucleotide substitutions per site was estimated for each pair, as described by Nei and Gojobori (1986), using the Jukes–Cantor formula. The parameters were calculated using Arlequin v3.5 and MEGA 11 (Tamura et al. 2021). A distance matrix based on the neighbor-joining (NJ) method was used to construct a *BoLA-DRB3* allele tree using the β1 domain nucleotide sequence of all alleles, performed with MEGA 11. Significance of the branches was assessed through 500 bootstrap replicates.

A logo of the antigen-binding site (ABS) for each population was created using WebLogo 3 (Crooks et al. 2004) with the BLOSUM62 substitution matrix. The color scheme was based on the chemical properties of the amino acids, namely polar (G, S, T, Y, C; green), neutral (Q, N; purple), basic (K, R, H; blue), acidic (D, E; red), and hydrophobic (A, V, L, I, P, W, F, M; black). To investigate whether certain codon sites were under diversifying selection within Creole breeds and SibCu, a Bayesian method implemented in OmegaMap (Wilson and McVean 2006) was used to calculate the selection index (ω) at each amino acid site. This method accounted for intragenic recombination and did not assume a known fixed genealogy, thereby reducing the risk of false detection of positive sites.

### Breed/population relationship

To study the relationship among breeds based on *BoLA-DRB3* polymorphisms, two types of analyses were performed, namely multivariate and phylogenetic. Principal component analysis (PCA) was conducted to condense the genetic variation in the *BoLA-DRB3* gene. Allele frequencies were used for the PCA, following the method by Cavalli-Sforza et al. (1994) using PAST v4.03 software (Hammer et al. 2001). Additionally, Nei’s standard genetic distance (Ds; Nei 1972) was calculated from allele frequencies using the NJ algorithm. Confidence in the groupings was assessed through bootstrap resampling with 1000 replications. Genetic distances and phylogenetic trees were constructed with POPTREE2 software (Takezaki et al. 2010) and visualized using FigTree v1.4.4 (Rambaut 2018). PCA was conducted using the amino acid frequencies from the ABS. Alleles sharing the same amino acid pattern for pockets 1, 4, 6, 7, and 9 were grouped to estimate their frequencies.

The distribution of alleles across populations was represented using a Venn diagram created with the R package “VennDiagram” (http://cran.r-project.org/). A second plot was generated to display only high-frequency alleles (≥ 5%).

## Results

### Gene frequency and genetic diversity at allele level

**Fig. 1:**
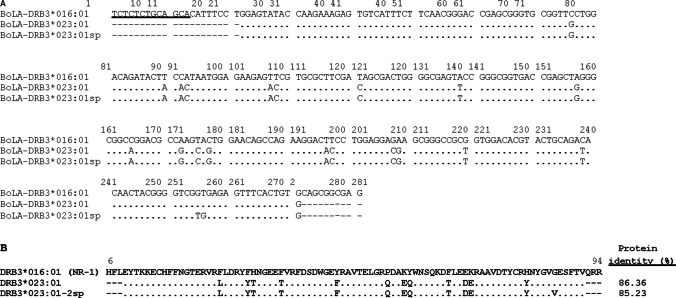
Alignment of the nucleotide (**a**) and predicted amino acid (**b**) sequences of the ß1 domains encoded by the novel *BoLA-DRB3* alleles detected in the Argentine Creole and Patagonian Argentine Creole cattle, showing their difference compared to the cDNA clone NR-1 (BoLA-DRB3*016:01 allele; Aida 1995). The reported *BoLA-DRB3* allele closer to the new variant was also included in the alignment (BoLA-DRB3*023:01). The nucleotide sequences reported were submitted to the IPD-MHC database and assigned the allele number BoLA-DRB3*023:02. The numbers indicate the positions of the amino acids in the mature protein. The amino acid residues are identical to those encoded by the BoLA-DRB3*016:01 cDNA clone

*BoLA-DRB3* exon 2 was genotyped using the PCR-SBT method, resulting in the identification of 60 reported alleles and one novel variant in the native populations studied. The new allele was found in both CrAr and CrArPat. The sequence was submitted to the IPD-MHC (https://www.ebi.ac.uk/ipd/mhc/group/BoLA), and the name assigned was BoLA-DRB3*023:02. It differed from BoLA-DRB3*023:01 by two single nucleotide polymorphisms at sites 255–256 (GT ˃ TG), resulting in a non-synonymous change [p.(G86V)]. The nucleotide and predicted amino acid sequences are shown in Fig. 1. The n_a_ ranged from 13 in CrArPat to 36 in LBC, the most polymorphic population (Table 1). Gene frequencies and cumulative gene frequencies within each breed are presented in Table 1 and Fig. S2, respectively. The number of high-frequency alleles (≥ 5%) varied from five to ten across the Latin American native populations studied till date (highlighted in bold, Table 2). Among them, BoLA-DRB3*015:01 and BoLA-DRB3*018:01 were the most common, being present in six populations, whereas the remaining high-frequency alleles were found in one to four breeds.

**Table 1:** Genetic diversity at allele

Population	***n***	**n**_**a**_	**h**_**o**_	**h**_**e**_	**FIS**	***p*-value**	**Slatkin neutrality test**
CrAr	107	16	0.869	0.871	0.002	0.178	0.076
CrArPat	22	13	0.818	0.820	0.003	0.472	0.796
HBC	61	26	0.885	0.945	0.064	0.001	0.058
LBC	122	36	0.910	0.952	0.044	0.499	0.007
PaCh	36	24	0.972	0.924	−0.052	0.016	0.747
SibCu	50	28	0.960	0.935	−0.027	0.790	0.643
CrLag	208	40	0.942	0.943	0.001	0.0004	0.212
HaVa	66	24	0.970	0.936	−0.036	0	0.126
AA	100	26	0.610	0.902	0.325	0	0.974
He	49	15	0.816	0.865	0.057	0.461	0.604
Sho	100	20	0.920	0.912	−0.009	0.110	0.064
HoAr	424	33	0.837	0.909	0.079	0.0002	0.396
HoPar	127	26	0.835	0.891	0.064	0.146	0.656
HoBo	159	22	0.930	0.898	−0.037	0.183	0.134
Gir	110	19	0.882	0.920	0.041	0.115	0.007
Ne	116	26	0.784	0.870	0.099	0.590	0.301
NeBh	195	33	0.760	0.855	0.113	0.014	0.442

Genetic diversity was calculated at the allele level for *BoLA-DRB3* in Latin American Creole cattle, European, and Zebu breeds using the following parameters: number of alleles (n_a_) and the observed (h_o_) and expected heterozygosity (h_e_). Moreover, Hardy-Weinberg Equilibrium measured through the F_IS_ index, and the Slatkin neutrality test was estimated to detect overdominant or balancing selection at *BoLA-DRB3*. Argentine Creole (CrAr); Patagonian Argentine Creole (CrArPat); Highland Bolivian Creole (HBC); Lowland Bolivian Creole (LBC); Paraguayan Pampa Chaqueño Creole (PaCh); Siboney (SibCu); Harton del Valle Creole (HaVa); Lageano Creole (CrLag); Angus (AA); Hereford (He); Shorthorn (Sho); Holstein from Argentina (HoAr), Bolivia (HoBo) and Paraguay (HoPa); Gir (Gir); Nelore (Ne); Nelore-Brahman (NeBh). *n* number of samples

**Table 2:** Gene frequencies of *BoLA-DRB3* alleles for each studied population

*BoLA-DRB3* allele	CrAr	CrPatAr	HBC	LBC	PaCh	SibCu	Bovine type	Reference
BoLA-DRB3*001:01	0.47	0	1.64	2.10	0	**9.00**	*Bos taurus*	Mikko & Anderson (1995)
BoLA-DRB3*002:01	1.87	0	4.92	7.40	**19.40**	2.00	*Bos taurus*	Mikko & Anderson (1995); Takeshima et al. (2011)
BoLA-DRB3*003:01	0	0	0.82	0	0	0	*Bos taurus*	Ammer et al. (1992); Mikko & Anderson (1995)
BoLA-DRB3*003:01:01	0	0	0.82	0	0	0	*Bos taurus*	Mikko & Anderson (1995)
BoLA-DRB3*005:01	0	**38.64**	0	3.70	0	0	*Bos taurus*	Mikko & Anderson (1995)
BoLA-DRB3*005:03	0	0	0	0	0	0	*Bos taurus*	Takeshima et al. (2011)
BoLA-DRB3*006:01	0	0	0	2.90	0	1.00	*Bos taurus*	Mikko & Anderson
BoLA-DRB3*007:01	0	0	4.10	**9.80**	0	1.00	*Bos indicus*; *Bos taurus*	Mikko & Anderson (1995); Takeshima et al. (2011)
BoLA-DRB3*007:02	0	0	0.82	0	0	0	*Bos indicus*	De & Singh (2006)
BoLA-DRB3*007:04	0	0	0	1.2	0	0	*Bos indicus*	Salim et al. (2021)
BoLA-DRB3*008:01	0	0	0	0	11.1	0	*Bos taurus*	Mikko & Anderson (1995); Takeshima et al. (2011)
BoLA-DRB3*008:02	0	0	0	0	0	0	*Bos indicus*	Salim et al. (2021)
BoLA-DRB3*009:01	0	0	0	4.10	2.78	**5.00**	*Bos taurus*	Mikko & Anderson (1995); Takeshima et al. (2011)
BoLA-DRB3*009:02	0	2.27	**7.38**	**7.80**	0	**8.00**	*Bos indicus*;* Bos taurus*	Xu et al. (1993); Mikko & Anderson (1995); Takeshima et al. (2011)
BoLA-DRB3*010:01	0	0	3.28	2.90	0	2.00	*Bos taurus*	Mikko & Anderson (1995); Takeshima et al. (2011)
BoLA-DRB3*010:02	0	0	0	0	**9.72**	0	*Bos indicus*; *Bos taurus*	Mikko & Anderson (1995); Takeshima et al. (2011)
BoLA-DRB3*011:01	**11.20**	2.27	3.28	3.70	1.39	**8.00**	*Bos indicus*;* Bos taurus*	Xu et al. (1993); Mikko & Anderson (1995); Takeshima et al. (2011)
BoLA-DRB3*011:04	0	0	0	4.50	0	0	*Bos indicus*	Salim et al. (2021)
BoLA-DRB3*012:01	1.87	0	**7.38**	3.30	0	3.00	*Bos indicus*; *Bos taurus*	Mikko & Anderson (1995); Takeshima et al. (2011)
BoLA-DRB3*013:01	0	0	0	1.20	0	0	*Bos taurus*; *Bos indicus*	Ammer et al. (1992); Mikko & Anderson (1995); Takeshima et al. (2011)
BoLA-DRB3*013:02	0	0	0	0	0	0	*Bos taurus*	Takeshima et al. (2011)
BoLA-DRB3*014:01:01	1.87	0	**5.74**	**7.8**	2.78	1	*Bos taurus*; *Bos indicus*	Mikko & Anderson (1995); Takeshima et al. (2011)
BoLA-DRB3*015:01	**5.14**	2.27	**6.56**	4.10	**11.10**	**7.00**	*Bos indicus*; *Bos taurus*	Gelhaus et al. (1995); Mikko & Anderson (1995); Takeshima et al. (2011)
BoLA-DRB3*015:06	0	0	0	0	0	0	*Bos indicus*	Direct submission
BoLA-DRB3*016:01	0	0	3.28	4.10	0	3.00	*Bos indicus*; *Bos taurus*	Gelhaus et al. (1995); Mikko & Anderson (1995); Takeshima et al. (2011)
BoLA-DRB3*017:01	0	0	**10.70**	1.60	1.39	0	*Bos indicus*; *Bos taurus*	Mikko & Anderson (1995)
BoLA-DRB3*017:03	0	0	0	0	0	0	*Bos indicus*	Maillard et al. (1999)
BoLA-DRB3*018:01	**7.01**	**6.82**	**12.30**	**9.40**	**5.56**	1.00	*Bos indicus*; *Bos taurus*	Ammer et al. (1992); Mikko & Anderson (1995)
BoLA-DRB3*018:04	0	0	0	0	0	0	*Bos indicus*	Salim et al. (2021)
BoLA-DRB3*018:06	0	0	0	0	0	1.00	*Bos indicus*	Direct submission
BoLA-DRB3*019:01	0	0	0.82	0	0	1.00	*Bos taurus*	Mikko & Anderson
BoLA-DRB3*020:01:01	0	0	0	0	0	0	*Bos indicus*	Mikko & Anderson (1995)
BoLA-DRB3*020:01:02	**26.2**	0	3.28	0.4	**5.56**	6	*Bos taurus*	Gelhaus et al. (1995)
BoLA-DRB3*020:02	0	0	2.46	0	0	0	*Bos indicus*; *Bos taurus*	Mikko & Anderson (1995); Takeshima et al. (2011)
BoLA-DRB3*020:03	0	0	0	0	0	1.00	*Bos indicus*	Maillard et al. 1999
BoLA-DRB3*020:06	0	0	0	0	0	0		
BoLA-DRB3*021:01	**7.48**	0	0.82	0.4	4.17	0	*Bos indicus*	Russell et al. (1994); Gelhaus et al. (1995); Mikko & Anderson (1995)
BoLA-DRB3*021:02	0	0	0	0	1.39	0	*Bos indicus*	Salim et al. (2021)
BoLA-DRB3*022:01	0	0	0.82	1.60	1.39	**8.00**	*Bos indicus*; *Bos taurus*	Russell et al. (1994); Gelhaus et al. (1995); Mikko & Anderson (1995); Takeshima et al. (2011)
BoLA-DRB3*022:12	0	0	0	0	0	0	*Bos indicus*	Salim et al. (2021)
BoLA-DRB3*022:14	0	0	0	0	0	0	*Bos indicus*	Salim et al. (2021)
BoLA-DRB3*023:01	3.74	**6.82**	0	0	**5.56**	0	*Bos indicus*	Mikko & Anderson (1995)
BoLA-DRB3*023:01_2sp	**15.9**	**6.82**	0	0	0	0		This work
BoLA-DRB3*024:06	0	0	0	0	0	0		
BoLA-DRB3*025:01:01	0	0	0	0	2.78	0	*Bos indicus*	Mikko & Anderson 1995
BoLA-DRB3*025:02	0	0	0	0.80	0	0	*Bos taurus*	Takeshima et al. 2001, 2003
BoLA-DRB3*026:01	0	0	0	0	0	1.00	*Bos indicus*; *Bos taurus*	Mikko & Anderson (1995); Takeshima et al. (2011)
BoLA-DRB3*026:06	0	0	0	0	0	0		
BoLA-DRB3*027:03	0	0	3.28	1.60	0	1.00	*Bos indicus*; *Bos taurus*	Ammer et al. (1992); Sharif et al. (2003)
BoLA-DRB3*027:05_sp	0	0	0	0	0	0		Casa et al
BoLA-DRB3*027:10	0	0	0	1.60	0	0	*Bos taurus*	Takeshima et al. (2011)
BoLA-DRB3*028:01	0	**15.91**	0.82	1.20	1.39	0	*Bos indicus*	Gelhaus et al. (1995); Takeshima et al. (2011)
BoLA-DRB3*028:02	3.74	0	0	0.8	1.39	1	*Bos taurus*	Takeshima et al. (2011)
BoLA-DRB3*028:04	0	2.27	0	0	0	0	*Bos indicus*	Salim et al. (2021)
BoLA-DRB3*029:01	0	0	1.64	0	0	0	*Bos indicus*	Gelhaus et al. (1995)
BoLA-DRB3*029:02	**9.35**	0	**6.56**	0.8	0	2.00	*Bos taurus*	Giovambattista et al. (2013)
BoLA-DRB3*030:01	0	0	0	0.8	1.39	**17.00**	*Bos indicus*; *Bos taurus*	Gelhaus et al. (1995)
BoLA-DRB3*031:01	0	0	1.64	1.60	0	0	*Bos taurus*	Sitte et al. (1995); Takeshima et al. (2011)
BoLA-DRB3*032:01	0	0	0	0	1.39	0	*Bos taurus*	Burke et al. (1991)
BoLA-DRB3*032:01_sp	0	0	0	0	0	0		Casa et al
BoLA-DRB3*032:02	0	0	0	0.40	1.39	0	*Bos taurus*	Sitte et al. (1995); Takeshima et al. (2011)
BoLA-DRB3*033:01	0	0	0	2.10	0	0	*Bos taurus*	Sitte et al. (1995)
BoLA-DRB3*034:01	0	0	0	0	0	1.00	*Bos taurus*	Sitte et al. (1995); Takeshima et al. (2011)
BoLA-DRB3*035:01	0	0	0	0.80	0	2.00	*Bos indicus*; *Bos taurus*	Maillard et al. (1999); Takeshima et al. (2011)
BoLA-DRB3*036:01	0	0	0	0.80	0	**5.00**	*Bos indicus*; *Bos taurus*	Maillard et al., Takeshima et al
BoLA-DRB3*037:01	0	**6.82**	0	0.8	2.78	0	*Bos primigenius*	Direct submission
BoLA-DRB3*039:01	0	0	0	0.4	0	0	*Bos primigenius*	Direct submission
BoLA-DRB3*044:01	0	4.55	0	0	1.39	0	*Bos taurus*	Takeshima et al. (2011)
BoLA-DRB3*045:01	0	0	0	0	0	1	*Bos taurus*	Direct submission
BoLA-DRB3*048:01	0	0	0.82	0	0	0	*Bos indicus*	De & Singh (2006)
BoLA-DRB3*048:02	3.27	2.27	4.10	1.2	1.39	0	*Bos taurus*	Takeshima et al. (2011)
BoLA-DRB3*049:01	0	0	0	0	0	1	*Bos indicus*	De & Singh (2006)
BoLA-DRB3*057:01	0	0	0	0	0	0	*Bos taurus*	Wang et al. (2008)
BoLA-DRB3*057:02	0	0	0	0	1.39	0	*Bos indicus*; *Bos taurus*	Wang et al. (2008)
BoLA-DRB3*067:01	0.47	0	0	0	0	0	*Bos indicus*	Direct submission
BoLA-DRB3*098:01	0.47	0	0	0	0	0	*Bos indicus*	Salim et al. (2021)
BoLA-DRB3*100:01	0	0	0	0	0	0	*Bos indicus*	Salim et al. (2021)
BoLA-DRB3*137:01	0	2.27	0	0	0	0	*Bos indicus*	Salim et al. (2021)

High-frequency alleles ($\ge 5\%$) are highlighted in bold. Bovine type and its reference where alleles were reported are detailed. Argentine Creole (CrAr); Patagonian Argentine Creole (CrArPat); Highland Bolivian Creole (HBC); Lowland Bolivian Creole (LBC); Paraguayan Pampa Chaqueño Creole (PaCh); Siboney (SibCu)

The large number of alleles within populations ($\ge 13\%$) resulted in h_o_ and h_e_ values exceeding 0.81 (Table 1). HWE analysis indicated deviations from theoretical proportions (*p *< 0.05) for only HBC and PaCh, due to a deficit and excess of heterozygote genotypes, respectively. To assess selection at the gene frequency level, the Slatkin neutrality test was applied; only LBC showed an even allele distribution, consistent with balanced selection (*p* = 0.007; Table 1). Comparison of the observed and expected gene frequencies for SibCu, based on its theoretical breed composition ($^{{~}^{5}\!\left/ \!\!{~}_{8}\right.}$ Holstein and $^{{~}^{3}\!\left/ \!\!{~}_{8}\right.}$ zebuine; CENCOP 2011), revealed that most alleles had almost no difference (Table S3). However, some alleles deviated from the expected proportions, showing either an increase or decrease in gene frequencies. Notably, the most abundant variant in SibCu, BoLA-DRB3*030:01, had low gene frequencies in both the parental breeds.

### Genetic diversity at DNA and amino acid sequence levels

**Fig. 2:**
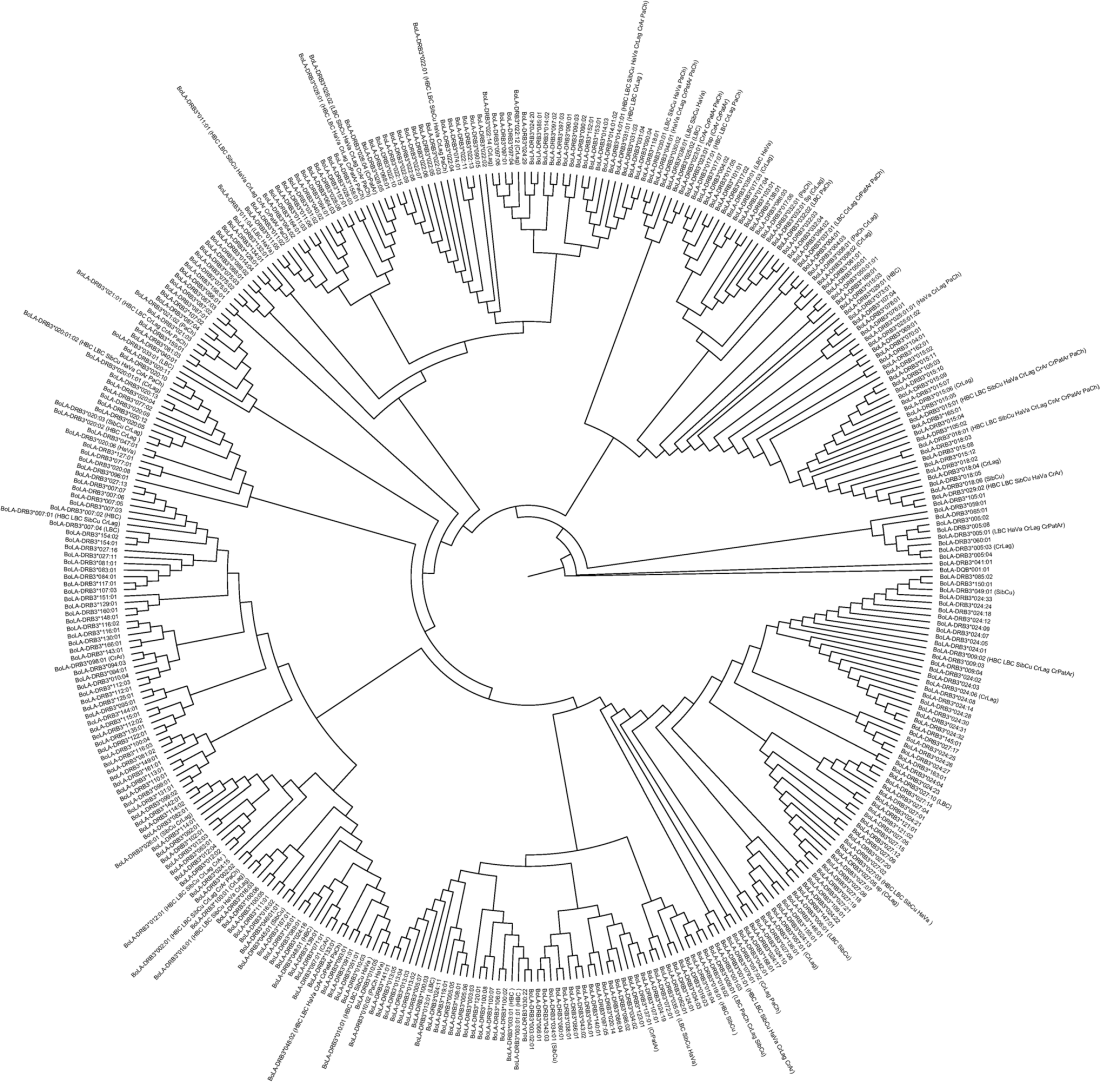
Neighbor-joining (NJ) tree constructed from the 270-bp nucleotide sequence including the β1 domain encoded by all reported *BoLA-DRB3* alleles and the novel one. Argentine Creole (CrAr); Patagonian Argentine Creole (CrArPat); Highland Bolivian Creole (HBC); Lowland Bolivian Creole (LBC); Paraguayan Pampa Chaqueño Creole (PaCh); Siboney (SibCu); Harton del Valle Creole (HaVa); Lageano Creole (CrLag); Angus (AA); Hereford (He); Shorthorn (Sho); Holstein from Argentina (HoAr), Bolivia (HoBo), and Paraguay (HoPa); Gir (Gir); Nelore (Ne); and Nelore-Brahman (NeBh)

Genetic diversity was assessed at the DNA level using two parameters: π and NPD. In native populations, π ranged from 0.073 in CrAr to 0.081 in the composite breed SibCu, whereas NPD varied from 17.88 in CrArPat to 20.19 in SibCu (Table S3). The numbers d_N_ and d_S_ (nucleotide substitutions per site) were averaged over all sequence pairs within each group and estimated for the entire exon 2 sequence and the ABS. As expected, the ABS exhibited a higher number of substitutions, particularly d_N_ substitutions, with values of approximately 0.4, more than twice as high as d_S_ values (approximately 0.09; Table 3). An NJ tree was constructed using 270-base pair nucleotide sequences of all reported *BoLA-DRB3* alleles. This tree presents nine major clusters, each containing several *BoLA-DRB3* alleles. The alleles identified in each native breed under study were interspersed throughout the clusters (Fig. 2).

**Table 3:** The number of nonsynonymous substitutions (dN) and synonymous substitutions (dS) from averaging over all sequence pairs within each group, nucleotide diversity (π), and the number of pairwise differences (NPD) are shown for the entire sequence, and dN and dS are shown for ABS positions

Entire sequence						ABS				
Breed	dS	dN	dS—dN	NPD	π		Breed	dS	dN	dS-dN
HBC	0.0354 (± 0.0135)	0.0947 (± 0.0185)	− 0.0593 (± 0.0199)	19.16	0.077		HBC	0.162 (± 0.06)	0.4031 (± 0.0761)	− 0.2411 (± 0.0953)
LBC	0.0368 (± 0.0131)	0.0984 (± 0.0189)	− 0.0616 (± 0.0198)	19.82	0.079		LBC	0.175 (± 0.0568)	0.4143 (± 0.0823)	− 0.2393 (± 0.0963)
CrAr	0.0351 (± 0.014)	0.0978 (± 0.0198)	− 0.0627 (± 0.0196)	18.29	0.073		CrAr	0.2118 (± 0.0689)	0.4304 (± 0.0902)	− 0.2186 (± 0.1003)
CrPatAr	0.0363 (± 0.0133)	0.1058 (± 0.0207)	− 0.0695 (± 0.0209)	17.88	0.076		CrPatAr	0.1383 (± 0.064)	0.4158 (± 0.091)	− 0.2776 (± 0.0972)
PaCh	0.0311 (± 0.0112)	0.0992 (± 0.0192)	− 0.0681 (± 0.0195)	19.14	0.077		PaCh	0.1499 (± 0.0481)	0.4093 (± 0.0856)	− 0.2594 (± 0.0937)
CrLag	0.0412 (± 0.0135)	0.0954 (± 0.0188)	− 0.0542 (± 0.0194)	20.38	0.079		CrLag	0.1497 (± 0.0501)	0.4053 (± 0.0842)	− 0.2556 (± 0.0901)
HaVa	0.0313 (± 0.0106)	0.1009 (± 0.0197)	− 0.0696 (± 0.0199)	19.87	0.079		HaVa	0.1209 (± 0.0496)	0.4008 (± 0.0878)	− 0.2799 (± 0.0909)
SibCu	0.035 (± 0.0135)	0.0999 (± 0.0194)	− 0.0649 (± 0.0205)	20.19	0.081		SibCu	0.1511 (± 0.0532)	0.4267 (± 0.0842)	− 0.2756 (± 0.0955)
AA	0.0372 (± 0.0139)	0.0967 (± 0.0186)	− 0.0594 (± 0.0203)	19.17	0.077		AA	0.1905 (± 0.06)	0.4255 (± 0.0834)	− 0.235 (± 0.0999)
He	0.033 (± 0.0141)	0.0978 (± 0.0185)	− 0.0648 (± 0.0208)	17.41	0.070		He	0.1657 (± 0.0572)	0.406 (± 0.0814)	− 0.2403 (± 0.0972)
Sho	0.0409 (± 0.0142)	0.0969 (± 0.0198)	− 0.056 (± 0.0206)	19.81	0.079		Sho	0.1754 (± 0.0568)	0.4334 (± 0.089)	− 0.258 (± 0.1026)
HoAr	0.0362 (± 0.0128)	0.0976 (± 0.0193)	− 0.0614 (± 0.0199)	20.17	0.078		HoAr	0.1475 (± 0.0494)	0.4086 (± 0.0857)	− 0.2611 (± 0.0953)
HoBo	0.0389 (± 0.0133)	0.1064 (± 0.02)	− 0.0675 (± 0.0202)	19.43	0.078		HoBo	0.1799 (± 0.0555)	0.4337 (± 0.0906)	− 0.2538 (± 0.1006)
HoPar	0.0359 (± 0.0139)	0.0993 (± 0.0192)	− 0.0634 (± 0.0207)	20.09	0.077		HoPar	0.1566 (± 0.0554)	0.4286 (± 0.0815)	− 0.2719 (± 0.0965)
Gir	0.038 (± 0.0145)	0.0958 (± 0.0193)	− 0.0577 (± 0.02)	19.47	0.078		Gir	0.2684 (± 0.0876)	0.4593 (± 0.0889)	− 0.1908 (± 0.1161)
Ne	0.0352 (± 0.0129)	0.0974 (± 0.0189)	− 0.0622 (± 0.0199)	19.54	0.070		Ne	0.1569 (± 0.0513)	0.4095 (± 0.0791)	− 0.2526 (± 0.0932)
NeBh	0.0389 (± 0.0135)	0.0973 (± 0.0191)	− 0.0584 (± 0.0201)	16.95	0.068		NeBh	0.1838 (± 0.0586)	0.4075 (± 0.084)	− 0.2237 (± 0.1002)

Argentine Creole (CrAr); Patagonian Argentine Creole (CrArPat); Highland Bolivian Creole (HBC); Lowland Bolivian Creole (LBC); Paraguayan Pampa Chaqueño Creole (PaCh); Siboney (SibCu); Harton del Valle Creole (HaVa); Lageano Creole (CrLag); Angus (AA); Hereford (He); Shorthorn (Sho); Holstein from Argentina (HoAr), Bolivia (HoBo), and Paraguay (HoPa); Gir (Gir); Nelore (Ne); and Nelore-Brahman (NeBh)

ABS logo schemes were created for each individual population and grouped by origin in native (Creole and SibCu), European (AA, He, HoAr, HoBo, HoPar, and Sho), and zebuine (Gir, Ne, and NeBh) populations (Fig. S3). The representations revealed similar patterns across breeds and groups. Although no variation was observed in amino acids at the pocket positions, differences in amino acid frequencies were evident. For instance, position 28 (pocket 7) showed a higher frequency of histidine (H) exclusively in native breeds. Variations in abundance were also observed at positions 11, 13, 30, 57, and 71. When comparing the composite breed SibCu with its ancestral parental populations, most patterns resembled those of Holstein (Fig. S3). The comparison of ABS motifs between European (AA, He, HoAr, HoBo, HoPar, and Sho) and Zebu (Gir, Ne, and NeBh) populations revealed variations across all pockets. The most notable difference was at position 86 (pocket 1), where glycine (G) was predominant in European populations, while valine (V) was more common in zebuine populations (Fig. S3).

The ω at each amino acid site was calculated to evaluate the presence of diversifying selection (ω > 1) across *BoLA-DRB3* exon 2 for Creole populations and SibCu. The analyses revealed high ω values at more than 25 sites in each group, with the most prominent peaks occurring in the ABS (Fig.S4).

### Breed/population relationship

**Fig. 3:**
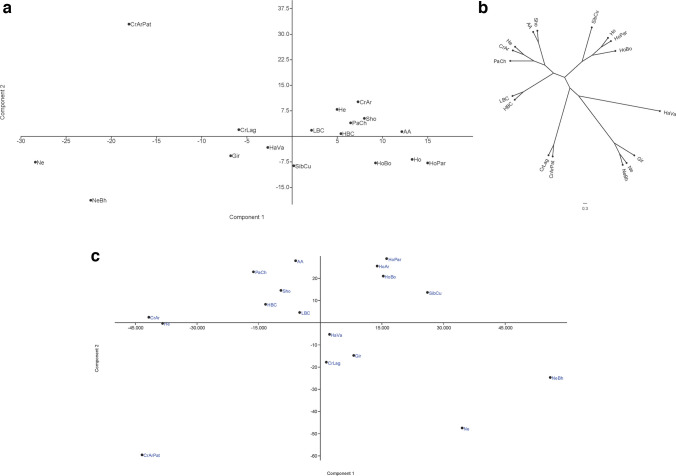
Principal component analysis (PCA; **a**); neighbor-joining tree representation of Nei’s genetic distance (**b**); and PCA conducted using the amino acid frequencies from the ABS (**c**). Argentine Creole (CrAr); Patagonian Argentine Creole (CrArPat); Highland Bolivian Creole (HBC); Lowland Bolivian Creole (LBC); Paraguayan Pampa Chaqueño Creole (PaCh); Siboney (SibCu); Harton del Valle Creole (HaVa); Lageano Creole (CrLag); Angus (AA); Hereford (He); Shorthorn (Sho); Holstein from Argentina (HoAr), Bolivia (HoBo), and Paraguay (HoPa); Gir (Gir); Nelore (Ne); and Nelore-Brahman (NeBh)

**Fig. 4:**
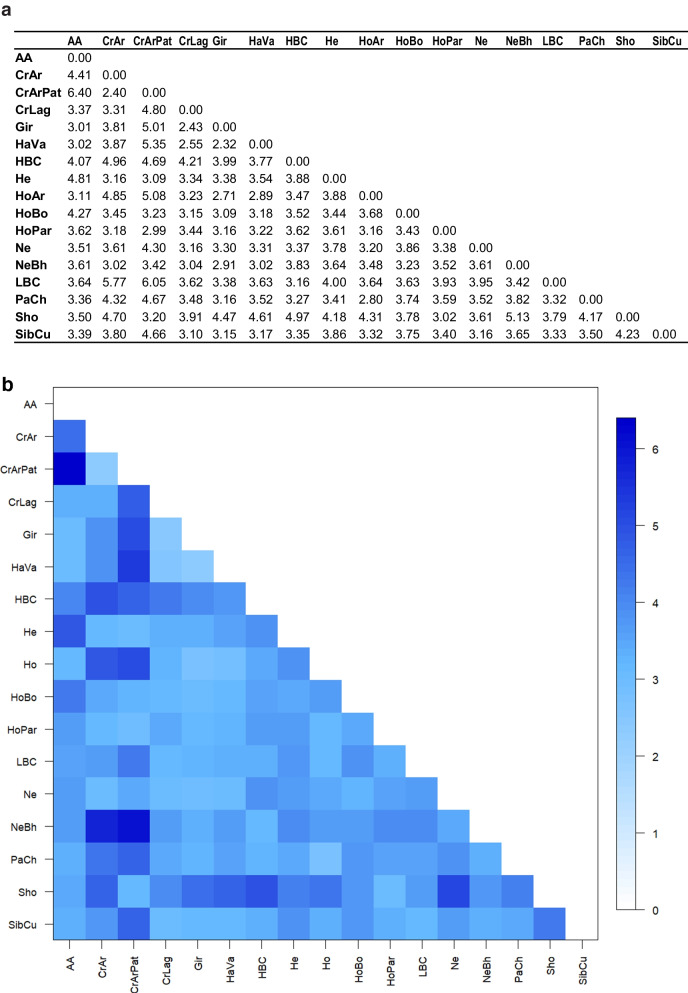
Genetic distance between pairs of populations estimated through Nei’s genetic distance (**a**) and heatmap graphic representation of the distances using an R-function: pairFstMatrix.r (**b**). Argentine Creole (CrAr); Patagonian Argentine Creole (CrArPat); Highland Bolivian Creole (HBC); Lowland Bolivian Creole (LBC); Paraguayan Pampa Chaqueño Creole (PaCh); Siboney (SibCu); Harton del Valle Creole (HaVa); Lageano Creole (CrLag); Angus (AA); Hereford (He); Shorthorn (Sho); Holstein from Argentina (HoAr), Bolivia (HoBo), and Paraguay (HoPa); Gir (Gir); Nelore (Ne); and Nelore-Brahman (NeBh)

The PCA revealed that the first principal component (PC1) accounted for 22.1% of the variance. In PC1, populations were distributed along the axis, with zebuine breeds at the extreme negative values and European taurine breeds at the opposite end. Creole populations and SibCu were positioned around the zero value. The distribution of Creole cattle was influenced by their degree of Zebu introgression, showing a trend towards either zebuine or taurine breeds. The second principal component (PC2) explained 17.2% of the genetic variance, primarily distinguishing CrArPat from the other populations (Fig. 3a). To address the significant impact of CrArPat on PC2, a PCA was conducted excluding this population. In this analysis, PC1 maintained the same pattern of population relationships with a similar genetic variance value (25.3%). PC2 accounted for 17.7% of the variance, showing clearer differentiation among Creole populations (Fig. S5). The NJ tree showed a distribution similar to that in the PCA, clearly differentiating the Zebu and taurine breeds (Fig. 3b). Creole populations clustered according to their historical and geographical distributions, with genetic distances detailed in Fig. 4. Additionally, PCAs based on the frequencies of ABS amino acid motifs—critical for epitope-antigen binding and immune response magnitude—did not reveal a distinct pattern across populations when analyzed by individual pockets. However, when all pockets were considered together, a similar distribution was observed across the breeds (Fig. 3c).

**Fig. 5:**
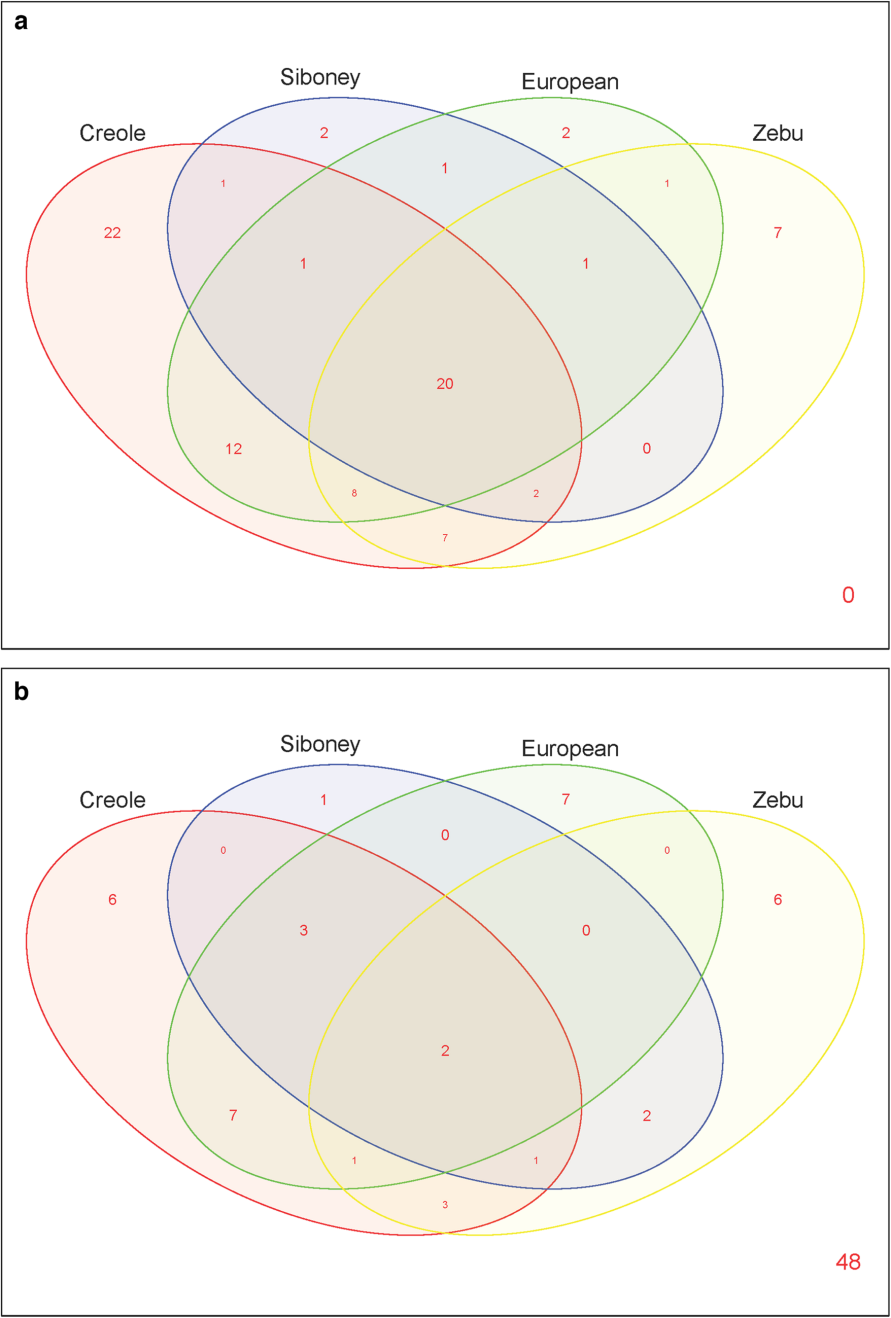
Venn diagram of *BoLA-DRB3* alleles (**a**) and high-frequency alleles (≥ 5%; **b**) shared by Creole populations (Argentine Creole, Patagonian Argentine Creole, Highland and Lowland Bolivian Creole, Paraguayan Pampa Chaqueño Creole, Harton del Valle Creole, and Lageano Creole), Siboney from Cuba (SibCu), European breeds (Angus, Hereford, Shorthorn, and Holstein), and Zebu breeds (Gir, Nelore, and Nelore-Brahman)

The Venn diagram illustrated the distribution of BoLA-DRB3 alleles in the four groups, namely Creole populations (from Argentina, Bolivia, Brazil, Colombia, and Paraguay), the composite breed Siboney from Cuba, Zebu breeds (Gir, Ne, and NeBh), and European breeds (AA, He, HoAr, HoBo, HoPar, and Sho). A total of 20 alleles were common to all the groups, with only two of high frequency (≥ 5%) (Fig. 5a–b). Notably, 22 alleles were exclusive to Creole populations, while the other groups had only seven unique alleles (Zebu) and two unique alleles each (Siboney and European). Additionally, Creole populations shared 12 alleles with European breeds, seven alleles with Zebu breeds, and eight alleles across all three groups.

## Discussion

The study of *BoLA-DRB3* has gained significant interest in recent decades due to its crucial role in immune response, specifically in antigenic peptide recognition and presentation, as well as its extraordinarily high degree of polymorphism. Genetic variability in *BoLA-DRB3* has been reported across various cattle breeds from different geographical regions (Miyasaka et al. 2011, 2012; Giovambattista et al. 2013, 2020a, b; Takeshima et al. 2014, 2015a, b, 2018; Bohorquez et al. 2020; Salim et al. 2021; Valenzano et al. 2022; Hamada et al. 2024). However, of the approximately 3000 cattle breeds recognized by the FAO (DAD-IS; https://www.fao.org/dad-is), only a few have been studied till date. The overall objective of this study was to characterize the genetic diversity of *BoLA-DRB3* in some native breeds from different countries of Latin America, thereby contributing to the global knowledge on this gene. The information obtained would allow us to address the following hypotheses: the Latin American native populations maintain a high degree of genetic variability and exhibit a distinct genetic profile for *BoLA-DRB3*; Creole populations within the same country may have diverged due to environmental adaptation; and the composite breed SibCu, with a theoretical proportion of $^{{~}^{5}\!\left/ \!\!{~}_{8}\right.}$-$^{{~}^{3}\!\left/ \!\!{~}_{8}\right.}$, may have undergone allele enrichment from one parent, driven by adaptation to tropical environments.

Over the last century, Creole cattle have been displaced and/or crossbred with introduced foreign breeds, leading to population fragmentation and reduction in their population size (Giovambattista et al. 2001; Michiels et al. 2023). This has resulted in reduced and isolated herds with low gene flow among Creole populations, causing the loss of alleles, especially those at low frequencies. To assess this, genetic diversity was evaluated at both gene frequency and DNA levels using various methods.

With the exception of CrArPat, all the studied populations exhibited n_a_ (≥ 15) comparable to those reported for other cattle breeds and high levels of genetic diversity (h_e_ ≥ 0.85). As published by Martinez et al. (2003), CrArPat exhibited a remarkably high-frequency allele, BoLA-DRB3*005:01 (> 30%), which was either absent or present at very low frequencies in other Creole breeds. This reduction in variability may be attributed to CrArPat being recovered from a feral herd in Los Glaciares National Park in southwest Patagonia, due to an isolation bottleneck event and/or because it is the only Creole population adapted to the southern cold forest. However, despite these variations in allele number and composition, the genetic diversity assessed at the molecular level showed π and NPD values to be similar across populations, including CrArPat, and comparable to those reported for other cattle breeds. The NJ tree for *BoLA-DRB3*, constructed with the β1 domain nucleotide sequence of all alleles, revealed that the detected variants were not in specific clusters but interspersed throughout the tree. This finding supported the ancient, trans-specific origin theory of MHC alleles (Klein 1987), which suggests that the genetic variants emerged before the domestication and speciation of cattle, resulting in their widespread presence across species and geographical regions. This hypothesis was further supported by the detection of various alleles (e.g., BoLA-DRB3*012:01, BoLA-DRB3*015:01, BoLA-DRB3*017:01, and BoLA-DRB3*018:01) in the studied populations, which are also described in other *Bos *sp., such as Indonesian *B. javanicus* (Agina et al. 2023). Similar findings have been reported for other native cattle breeds (Giovambattista et al. 2013, 2020a; Salim et al. 2021; Hamada et al. 2024). Additionally, the presence of European and African ancestry, as well as some degree of Zebu introgression in Latin American Creole cattle germplasm (Liron et al. 2006), may contribute to and explain the observed genetic variability at both allele and DNA levels.

Although Creole populations did not form a narrow cloud in the PCA or a distinct cluster in the phylogenetic tree, they tended to be positioned between the European taurine and zebuine breeds. This positioning might be due to their unique allele combinations and gene frequencies, as indicated by the Venn diagram, which showed that 22 of the detected alleles were exclusive to native populations. The historical origin and environmental adaptation of Creole cattle and SibCu may have contributed to this distinctive *BoLA-DRB3* diversity. Reported studies and databases allowed us to infer the private origin of these identified alleles. Both BoLA-DRB3*023:01_2sp and BoLA-DRB3*029:02 alleles were first detected in Creole cattle, with the latter recently identified in the African native cattle breed Baggara (Salim et al. 2021). Additionally, BoLA-DRB3*028:01, previously reported in Boran (Russell et al. 1994; Gelhaus et al. 1995), was found to be abundant in CrLag, HaVa, and CrArPat. Previous studies had identified an African component in the genome of Latin American Creole cattle, based on mitochondrial DNA and Y chromosome haplotypes (Ginja et al. 2019). This African genetic component originated from native Iberian cattle, the ancestors of Creole cattle, and may also have been directly contributed by African cattle introduced via the transatlantic slave trade routes (Ginja et al. 2019). Unfortunately, as only the Morucha Spanish breed has been studied for this gene, the magnitude of contribution from these proposed origins remains unclear (Bohorquez et al. 2020). The rapid adaptation of the introduced Iberian cattle to the tropical and subtropical conditions of the American continent may have been influenced by this African genetic component. Recent studies reported that Zebu introgression in Latin American Creole cattle is higher in tropical and subtropical regions than in temperate and high-altitude environments (Giovambattista et al. 2000; Liron et al. 2006). Consequently, some alleles abundant in Zebu populations are frequently found in certain Creole populations. The BoLA-DRB3*022:01 allele exhibited a high frequency (> 5%) in native cattle with significant Zebu influence (SibCu, CrLag, and HaVa) and was notably abundant (> 20%) in Nelore and Nelore-Brahman. Additionally, the allele was reported in *Bos indicus* African breeds (Russell et al. 1994; Gelhaus et al. 1995; Mikko and Andersson 1995) and was found at high frequencies (> 5%) in native cattle from Myanmar and Sudan (Giovambattista et al. 2020a, b; Salim et al. 2021). In contrast, this allele was either absent or present at very low frequencies in European taurine breeds (Takeshima et al. 2014, 2015a, b; present work). Other alleles reported in *B. indicus* (Mikko & Anderson 1995; Maillard et al. 1999), such as BoLA-DRB3*020:03, BoLA-DRB3*021:01, and BoLA-DRB3*025:01:01, were found exclusively in native populations (CrAr, CrLag, HaVa, HBC, LBC, PaCh, and SibCu) and were identified in native cattle from Myanmar, Sudan, and Egypt (Giovambattista et al. 2020a; Salim et al. 2021; Hamada et al. 2023). This zebuine influence was evident in the PCA, where PC1 showed the distribution of native cattle relative to zebuine or taurine breeds, primarily according to their degree of Zebu introgression. In this context, the tropical native populations were located closer to the zebuine breeds.

Goszczynski et al. (2018) proposed that selection processes for the composite Brangus breed may have enriched the *BoLA* region with Brahman haplotypes to enhance adaptation to subtropical environments. In this regard, SibCu may be expected to have undergone a similar selection process to preserve the desirable traits of each parental breed. The estimated gene frequencies for SibCu, based on its theoretical breed proportions, showed that most alleles had expected and observed differences of less than 1%. The remaining variants exhibited enrichment for both parental breeds in a similar proportion. This observation was consistent with the logo pattern of SibCu, which resembled Holstein more closely than zebuine breeds. The results were consistent with the phylogenetic tree and PCA positions, showing SibCu to be closer to Holstein than to zebuine populations. Remarkably, two other alleles exhibited notable patterns; BoLA-DRB3*030:01, the most abundant, was rare in both parentals, and BoLA-DRB3*029:02 was exclusive to Creole cattle. The presence of this last variant in SibCu may be attributed to its historical development, where the zebuine component included local populations formed by crossbreeding imported Zebu individuals with Creole cattle (Utsunomiya et al. 2022). This absorption process of local native populations with foreign breeds has been common across various Latin American countries and was clearly observed in the matrilineages (Ginja et al. 2019).

The current results indicated that the studied native populations still maintain a high level of genetic diversity at the *BoLA-DRB3* gene despite the reduction in their population size. Different mechanisms have been proposed to explain this phenomenon, including overdominance and balancing selection. The significant excess of heterozygosity in MHC genes in mammal populations is often explained by the overdominance theory, which suggests that heterozygous individuals can recognize a wider range of foreign antigens, thereby enhancing their relative fitness, compared to homozygotes (Hedrick et al. 1991; Hughes and Nei 1989). In cattle, Takeshima et al. (2008) reported a significant deviation from HWE in the class II *BoLA-DQA1* gene in cows affected by mastitis caused by *Escherichia* or *Streptococcus* bacteria. Similarly, Lo et al. (2021b) analyzed the *BoLA-DRB3* gene in Japanese Holsteins and proposed a heterozygote advantage related to the outcomes of bovine leukemia virus infection. In this study, only two populations presented a deviation from HWE; PaCh exhibited an excess of heterozygotes, while HBC showed a deficit of heterozygotes. The deficit in HBC could be explained by the Wahlund effect, as this population comprises animals from small isolated farms. Similar results were observed in other cattle breeds studied previously (CrLag, HoAr, and NeBh). To evaluate the balancing selection hypothesis, Slatkin’s exact test and sequence-level statistical methods were employed. The gene frequency profile of *BoLA-DRB3* in Creole cattle showed an even distribution in LBC (*p* = 0.007), indicating balancing selection. A similar distribution was observed in other cattle breeds including Bolivian Gyr, Japanese Black, Pyer Sein, and Shwe Ni (Takeshima et al. 2018; Giovambattista et al. 2020a, b). Conversely, balancing selection was not detected in other Creole cattle populations (*p* > 0.025), despite having many alleles with similar frequencies. The results aligned with the observations from most cattle breeds analyzed till date.

Selection at both nucleotide and amino acid levels was evaluated. As expected, the rates of d_s_ and d_n_ were higher at the ABS than in the entire sequence. Analysis of the codon sites under selection in Creole cattle and SibCu revealed that the most prominent peaks (ω > 40) were at positions 11, 13, 37, and 74. These sites corresponded to regions with higher amino acid variability observed in the ABS Logos. For example, position 37, associated with pocket 9, exhibited five different amino acids, suggesting that selection at these sites may drive a broader amino acid repertoire.

The ABS logo schemes were created for populations and in-groups, including native (Creole and SibCu), European (AA, He, HoAr, HoBo, HoPar, and Sho), and zebuine (Gir, Ne, and NeBh). Remarkably, variations in amino acid frequencies were observed in multiple positions when comparing Creole populations from the same country but adapted to different environments (CrAr and CrArPat from Argentina, and HBC and LBC from Bolivia). These variations were mainly observed in Argentinian Creoles, where 14 changes affected all pockets. In contrast, Bolivian Creoles showed variations in pockets 4, 6, 7, and 9. The presence of specific amino acid motifs at ABS of the *BoLA-DRB3* gene affects the magnitude and epitope specificity of antigen-specific T-cell responses and is crucial for antigenic presentation and the onset of the immune response. Also, this gene has previously been associated with resistance or susceptibility to certain diseases (Yoshida et al. 2009; Lo et al. 2021a).

## Conclusion

The present results demonstrated that the Latin American native populations exhibit high variability in the *BoLA-DRB3* gene, including private alleles, and a distinct genetic profile. The characteristics may have resulted from their origin, subsequent selection, and environmental adaptation. The distinctive features of these populations highlight their value as a zoo-genetic resource and emphasize the importance of their conservation.

## Supplementary Information

Below is the link to the electronic supplementary material.

ESM1 Fig. S1 Sampling sites of Argentine Creole and Patagonian Argentine Creole (red); Bolivian Creole from Cochabamba Department and Bolivian Saavedreño Creole (blue); Paraguayan Pampa Chaqueño Creole (grey); and Siboney (purple)(PNG 60.4 KB)

ESM2 Fig. S2 Cumulative gene frequency distribution of BoLA-DRB3 alleles in Argentine Creole (CrAr); Patagonian Argentine Creole (CrArPat); Highland Bolivian Creole (HBC); Lowland Bolivian Creole (LBC); Paraguayan Pampa Chaqueño Creole (PaCh); Siboney (SibCu); Harton del Valle Creole (HaVa); and Lageano Creole (CrLag) (PNG 51.0 KB)

ESM3 Fig. S3 Logos of the antigen-binding site (ABS) for each population created using WebLogo 3 (Crooks et al. 2004) with the BLOSUM62 substitution matrix. The amino acids are ordered according to their positions in the following pockets: pocket 1 (pos. 85, 86, 89, and 90), pocket 4 (pos. 13, 26, 28, 70, 74, and 78), pocket 6 (pos. 11 and 30), pocket 7 (pos. 28, 47, 61, 67, and 71), and pocket 9 (pos. 9, 37, and 57). The color scheme was based on the chemical properties of the amino acids; polar (G, S, T, Y, C; green), neutral (Q, N; purple), basic (K, R, H; blue), acidic (D, E; red), and hydrophobic (A, V, L, I, P, W, F, M; black). Argentine Creole (CrAr); Patagonian Argentine Creole (CrArPat); Highland Bolivian Creole (HBC); Lowland Bolivian Creole (LBC); Paraguayan Pampa Chaqueño Creole (PaCh); Siboney (SibCu); Harton del Valle Creole (HaVa); Lageano Creole (CrLag); Angus (AA); Hereford (He); Shorthorn (Sho); Holstein from Argentina (HoAr), Bolivia (HoBo), and Paraguay (HoPa); Gir (Gir); Nelore (Ne); and Nelore-Brahman (NeBh)(PNG 369 KB)

ESM4 Fig. S4 Estimated values of the selection index (ω) in each amino acid site along BoLA-DRB3 exon 2 in Creole cattle populations (Argentine Creole, Patagonian Argentine Creole, Highland Bolivian Creole, Lowland Bolivian Creole, Paraguayan Pampa Chaqueño Creole, Harton del Valle Creole, Lageano Creole; blue) and SibCu breed (orange) (PNG 52.9 KB)

ESM5 Fig. S5 Principal Components Analysis (PCA) excluding the Patagonian Argentine Creole population to minimize its significant impact on the overall analysis. Argentine Creole (CrAr); Highland Bolivian Creole (HBC); Lowland Bolivian Creole (LBC); Paraguayan Pampa Chaqueño Creole (PaCh); Siboney (SibCu); Harton del Valle Creole (HaVa); Lageano Creole (CrLag); Angus (AA); Hereford (He); Shorthorn (Sho); Holstein from Argentina (HoAr), Bolivia (HoBo) and Paraguay (HoPa); Gir (Gir); Nelore (Ne); Nelore-Brahman (NeBh)(PNG 27.3 KB)

ESM6 (XLSX 11.0 KB)

ESM7 (DOCX 16.4 KB)

ESM8 (XLSX 42.4 KB)

## Data Availability

Data is provided within the manuscript or supplementary information files.
