# Supplementary figures and images for: Genetic diversity of BoLA-DRB3 in Latin American Creole cattle: an update of the state of the art
Source: Immunogenetics. 2025 Sep 29;77(1):28. doi: 10.1007/s00251-025-01384-w (PMC12717166; doi:10.1007/s00251-025-01384-w)

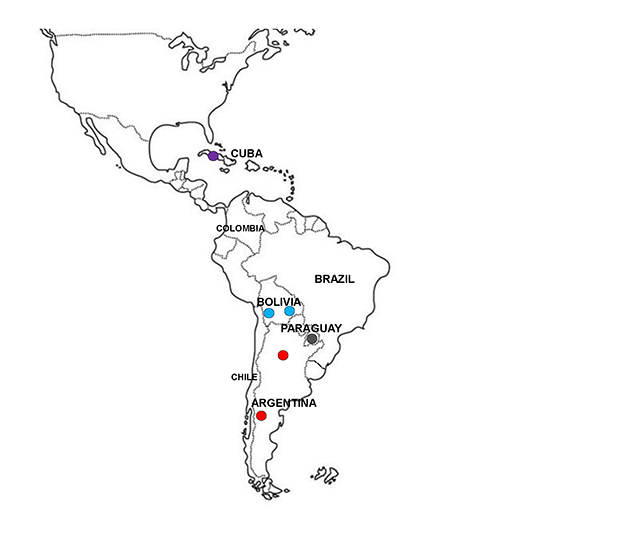

Supplement: Supplementary file 1 — Fig. S1 Sampling sites of Argentine Creole and Patagonian Argentine Creole (red); Bolivian Creole from Cochabamba Department and Bolivian Saavedreño Creole (blue); Paraguayan Pampa Chaqueño Creole (grey); and Siboney (purple)(PNG 60.4 KB) [file 251_2025_1384_MOESM1_ESM.png]

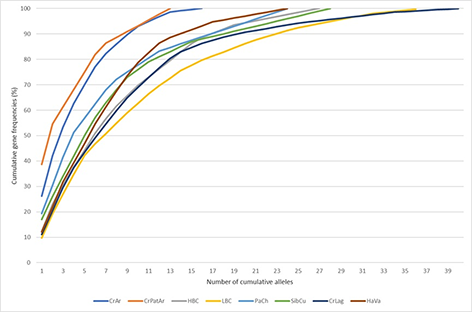

Supplement: Supplementary file 2 — Fig. S2 Cumulative gene frequency distribution of BoLA-DRB3 alleles in Argentine Creole (CrAr); Patagonian Argentine Creole (CrArPat); Highland Bolivian Creole (HBC); Lowland Bolivian Creole (LBC); Paraguayan Pampa Chaqueño Creole (PaCh); Siboney (SibCu); Harton del Valle Creole (HaVa); and Lageano Creole (CrLag) (PNG 51.0 KB) [file 251_2025_1384_MOESM2_ESM.png]

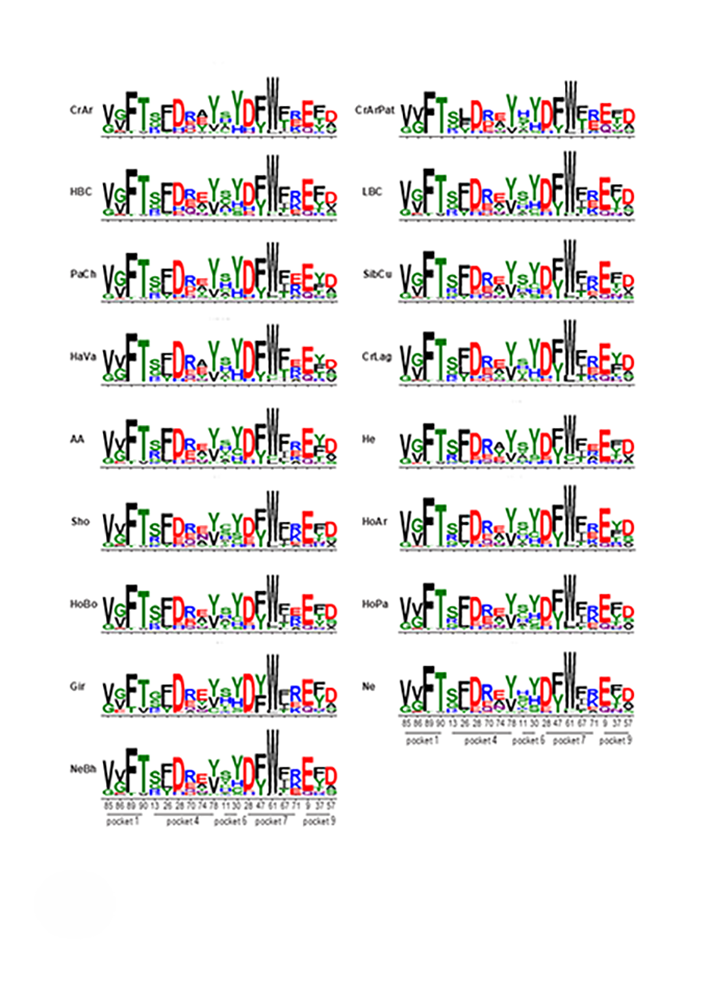

Supplement: Supplementary file 3 — Fig. S3 Logos of the antigen-binding site (ABS) for each population created using WebLogo 3 (Crooks et al. 2004) with the BLOSUM62 substitution matrix. The amino acids are ordered according to their positions in the following pockets: pocket 1 (pos. 85, 86, 89, and 90), pocket 4 (pos. 13, 26, 28, 70, 74, and 78), pocket 6 (pos. 11 and 30), pocket 7 (pos. 28, 47, 61, 67, and 71), and pocket 9 (pos. 9, 37, and 57). The color scheme was based on the chemical properties of the amino acids; polar (G, S, T, Y, C; green), neutral (Q, N; purple), basic (K, R, H; blue), acidic (D, E; red), and hydrophobic (A, V, L, I, P, W, F, M; black). Argentine Creole (CrAr); Patagonian Argentine Creole (CrArPat); Highland Bolivian Creole (HBC); Lowland Bolivian Creole (LBC); Paraguayan Pampa Chaqueño Creole (PaCh); Siboney (SibCu); Harton del Valle Creole (HaVa); Lageano Creole (CrLag); Angus (AA); Hereford (He); Shorthorn (Sho); Holstein from Argentina (HoAr), Bolivia (HoBo), and Paraguay (HoPa); Gir (Gir); Nelore (Ne); and Nelore-Brahman (NeBh)(PNG 369 KB) [file 251_2025_1384_MOESM3_ESM.png]

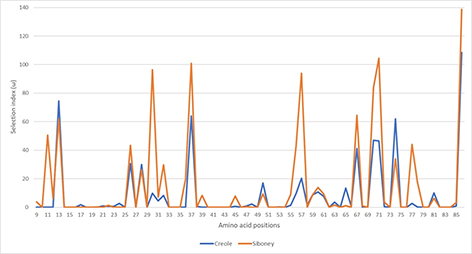

Supplement: Supplementary file 4 — Fig. S4 Estimated values of the selection index (ω) in each amino acid site along BoLA-DRB3 exon 2 in Creole cattle populations (Argentine Creole, Patagonian Argentine Creole, Highland Bolivian Creole, Lowland Bolivian Creole, Paraguayan Pampa Chaqueño Creole, Harton del Valle Creole, Lageano Creole; blue) and SibCu breed (orange) (PNG 52.9 KB) [file 251_2025_1384_MOESM4_ESM.png]

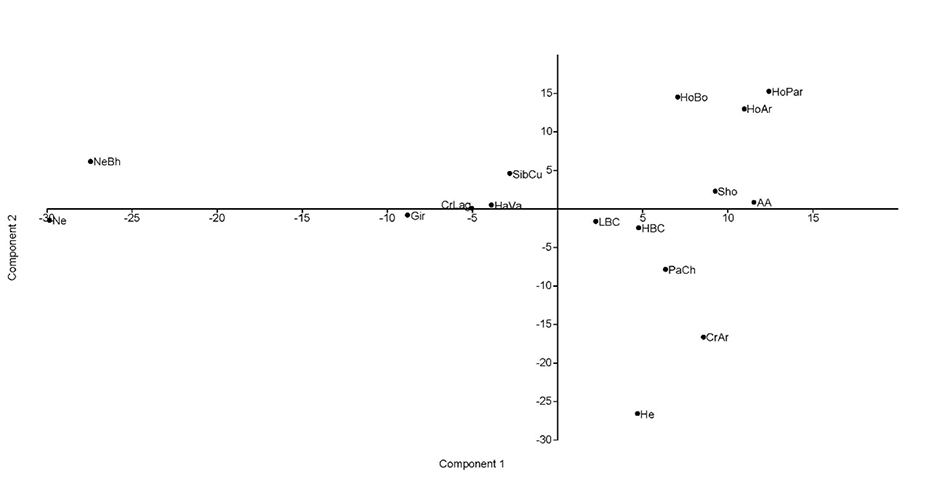

Supplement: Supplementary file 5 — Fig. S5 Principal Components Analysis (PCA) excluding the Patagonian Argentine Creole population to minimize its significant impact on the overall analysis. Argentine Creole (CrAr); Highland Bolivian Creole (HBC); Lowland Bolivian Creole (LBC); Paraguayan Pampa Chaqueño Creole (PaCh); Siboney (SibCu); Harton del Valle Creole (HaVa); Lageano Creole (CrLag); Angus (AA); Hereford (He); Shorthorn (Sho); Holstein from Argentina (HoAr), Bolivia (HoBo) and Paraguay (HoPa); Gir (Gir); Nelore (Ne); Nelore-Brahman (NeBh)(PNG 27.3 KB) [file 251_2025_1384_MOESM5_ESM.png]
