# Supplementary material for: Genetic diversity of BoLA-DRB3 in Latin American Creole cattle: an update of the state of the art
Source: Immunogenetics. 2025 Sep 29;77(1):28. doi: 10.1007/s00251-025-01384-w (PMC12717166; doi:10.1007/s00251-025-01384-w)
Supplement: Supplementary file 7 — (DOCX 16.4 KB) [file 251_2025_1384_MOESM7_ESM.docx]

**Table S2**. Primer information used for the *BoLA-DRB3* typing

| Primer | Sequence | Exon/intron location of individual primers | Accession number of the reference *BoLA-DRB3* gene sequence |
| --- | --- | --- | --- |
| DRB3FRW | 5’-CGCTCCTGTGAYCAGATCTATCC-3’ | intron | U78548 |
| DRB3REV | 5’-CACCCCCGCGCTCACC-3’ | intron |  |
